# Sealing Ability of Three Commercial Mineral Trioxide Aggregates and an Experimental Root-End Filling Material

**Published:** 2006-10-01

**Authors:** Saeed Asgary, Mohammad Jafar Eghbal, Masoud Parirokh, Hassan Torabzadeh

**Affiliations:** 1 *Department of Endodontics, Iranian Center for Endodontic Research, Dental School, Shahid Beheshti University of Medical Sciences, Tehran, Iran*; 2 *Department of Endodontics, Iran Center for Dental Research, Dental School, Shahid Beheshti University of Medical Sciences, Tehran, Iran*; 3 *Department of Endodontics, Dental School, Kerman University of Medical Sciences, Kerman, Iran*; 4 *Department of Dental Material Science, Dental School, Shahid Beheshti University of Medical Sciences, Tehran, Iran*

**Keywords:** Calcium Enriched Mixture, Microleakage, MTA, Root-End Filling

## Abstract

**INTRODUCTION:** The purpose of this study was to compare the sealability of three different commercial types of mineral trioxide aggregate (MTA) and calcium enrichment mixture (CEM) cement as an experimental root-end filling material.

**MATERIALS AND METHODS:** Forty-six single rooted teeth were cleaned, shaped, and obturated. The apical 3 mm of each root was resected and root-end cavities with 3 mm depth were prepared. The samples were randomly divided into 4 experimental groups comprised of 10 roots each. The cavities were filled with CEM cement and MTAs. Six roots were used as positive and negative controls. Samples were prepared and then immersed in 1% methylene blue dye for 3 days. Roots were split longitudinally and examined under stereomicroscope.

**RESULTS:** Positive and negative controls responded as expected. CEM cement showed the least mean dye penetration value. ANOVA revealed no statistically significant differences among experimental groups.

**CONCLUSION:** It was concluded that the experimental CEM cement exhibited similar sealing property as commercial types of MTA.

## INTRODUCTION

Endodontic surgery often includes the following steps: debridement and curettage of periradicular lesion from pathologic tissues, exposure of root apex, root-end resection, root-end preparation and insertion of a root-end filling material. The ideal root-end filling material seals the contents within the canal, preventing egress of any bacteria or toxic materials into the periradicular tissues (1).

Numerous materials have been proposed for this reason. An ideal root-end filling material should be biocompatible, antibacterial, nontoxic, non-corrosive, nonresorbable, dimensionally stable, easy to handle, unaffected by moisture, radiopaque, cost-effective, adaptable to the dentinal walls, and finally able to induce regeneration of the PDL complex, specifically cementogenesis over the root-end filling itself (1-2).

The sealability of root-end filling materials has been assessed by different methods such as dye/ ink (methylene blue dye, India ink, fluorescent and reactive blue dyes, eosin, basic fuschin, silver nitrate and gold-palladium) or bacterial/ endotoxin penetration (3-7), electrochemical methods (8), fluid filtration technique (9), radioisotope tracing (10), and evaluation of marginal adaptation by scanning electron microscopy (11).

Among the aforementioned methods dye penetration studies is the most commonly used technique for microleakage assessment of root-end filling materials (12).

Mineral trioxide aggregate (MTA) is a relatively new filling material made up of fine hydrophilic particles. During mixing the powder with water, hydration of the particles results in a colloidal gel, which solidifies to a hard structure in less than 4 hours (13).

MTA has several clinical applications in endodontics such as management of internal root resorption, one-step apexification, pulp capping, pulpotomy, repair of root and furcation perforations and root-end filling (13-14). When used as a root-end filling material, MTA stimulates the healing of periradicular tissues to almost normal condition (15). Over the last decade, numerous articles on the properties and applications of MTA have been published.

Authors have known the following commercial materials: 1- ProRoot MTA, gray colored cement (GMTA), 2- ProRoot MTA, a tooth colored formula (WMTA), 3- Angelus MTA (AMTA), and 4- Root MTA (RMTA).

GMTA may cause tooth discoloration particularly when it is used to cap or seal a perforation, where aesthetics is the priority. WMTA has been introduced in order to address this issue (16). The compositional differences of GMTA and WMTA have been reported (17). The detail of chemical composition of AMTA was reported by a Brazilian manufacture (18). It has been claimed that AMTA is the equivalent product to WMTA (19). RMTA has been recently introduced and, according to the Iranian manufacturer, has similar composition compared to ProRoot MTA. In one previous study, the authors substantiated manufacture claim showing only a slight difference between the amounts of major elements (20).

Although MTA has superior biocompatibility in comparison with traditional materials used in root-end filling procedure, but it has delayed setting time (13), poor handling characteristics (21), off-white color, and finally it is an expensive material. To decrease the expenses, Portland Cement (PC) has been compared with MTA in recent studies. It has been shown that PC contains the same composition as MTA, except bismuth oxide (22-23). Researchers have also reported similar biocompatibility (24) and suggested that PC has the potential to be used as an alternative material to MTA.

The advantages and disadvantages of GMTA, WMTA, RMTA and PCs have been documented (17,20,23,25) as prerequisites of a project on production of a new dental material that combines the superior biocompatibility of MTA with appropriate setting time, handling characteristics, chemical properties, color, and reasonable price. This experimental material or so-called calcium enriched mixture (CEM) cement was formulated using different calcium compounds.

The purpose of this in vitro study was to compare the sealing ability of different brands of mineral trioxide aggregate (MTA) and calcium enriched mixture (CEM) cement as a new root-end filling material by methylene blue dye penetration method.

## MATERIALS AND METHODS

Forty-six freshly extracted human teeth were used in this study. All procedures were carried out according to protocols approved by the Iranian Center for Dental Research, Dental School, Shahid Beheshti University of Medical Sciences in 2005.

The selection criteria were presence of a single root canal, no evidence of crack or fracture, root caries or resorption and previous endodontic treatment, root length of at least 14 mm, and an initial apical size no greater than # 30 K-file. Each tooth was decoronated using a cylindrical diamond bur (D&Z, Germany) at high-speed with water spray coolant. After enlarging the apical foramen up to K-File Size # 30 (Mani, Japan), root canals were instrumented at 0.5 mm from the apex up to K-File Size # 50, cleaned and shaped using standard step-back technique. The canals were copiously irrigated with 5.25% sodium hypochlorite solution during cleaning and shaping. After final irrigation, canals were dried with paper points (Ariadent, Iran) and obturated with laterally condensed gutta-percha (Ariadent, Iran) and Roth 801 root canal sealer (Roth International LTD- USA). After removing 2 to 3 mm of gutta-percha, the root access cavities were filled with Coltosol (Coltene, AG, Switzerland). The roots were stored in sterile normal saline and placed in a 37°C incubator for 48 h.

Root-end resections were made by removing 2 to 3 mm of the apex at a 90-degree angle to the long axis of the root with a cylindrical carbide bur (D&Z, Germany) using a high-speed with water spray coolant. The 3-mm deep class I root-end preparation were made using an ultrasonic power unit (miniPiezon, EMS, Nyon, Switzerland) with ultrasonic retrotips (DT-043, EMS, Nyon, Switzerland) and finally irrigated with 5.25% sodium hypochlorite, 17% ethylenediaminetetraacetic acid, and normal saline, respectively.

The cavities were dried with absorbent paper points and the 40 roots were randomly divided into four experimental groups, each comprised of ten roots. The root-end surfaces were observed under stereomicroscope before filling in order to discard the roots with crack on their surfaces.

In each group root-end cavities were filled with one of the following materials according to the manufacturers’ instruction: 1-ProRoot MTA, a Tooth Colored Formula (Dentsply Tulsa Dental, Tulsa, OK, USA), 2-Angelus MTA (Angelus Soluções Odontológicas, Londrina, PR, Brazil), 3-Root MTA (Salami far, Tehran, Iran), and 4-calcium enriched mixture (CEM) cement. The excess material was removed with wet cotton pellets and the roots were placed in 100% humidity at 37° C for 24 h.

The entire root surfaces, except for the area corresponding to the resected root-end surface were then coated with two layers of red nail varnish (Etude power lasting, Etude co, Seoul, Korea) and allowed to dry. Six roots were used as positive and negative controls. Three canals were filled with gutta-percha and no sealer so that were served as positive control group, and three additional canals were also filled in the same manner as group 1 and then completely covered with nail varnish to be served as negative controls.

The roots were then immersed in 1% methylene blue dye neutral solution and kept in an environment at 37 °C for 72 h. Following exposure to dye, the roots were rinsed in running water for 5 min, dried for 24 h at room temperature, grooved on the buccal and lingual surfaces with a # 5 diamond disc (DFS, Germany) and split in two fragments. Linear dye penetration was measured using a stereomicroscope (SZX9/12, Olympus, Tokyo, Japan) with a 0.1 mm ocular grid (U-OCMSQ10/10, Eyepiece Micrometer, Olympus) at x10 magnification. An examiner measured the extension of dye penetration between the root-end filling material and dentinal walls in the blind manner. The mean microleakage recorded for the experimental groups were analyzed statistically by One-way ANOVA test. Statistically significant differences among the groups were set at p<0.05.

## RESULTS

**Figure 1:**
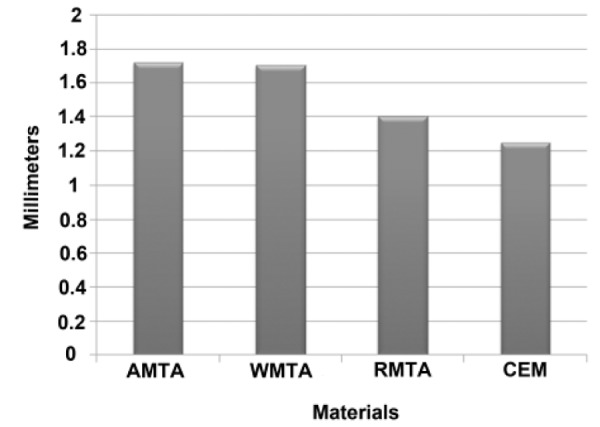
The mean dye microleakage values of the experimental groups (AMTA: Angelus-MTA, WMTA: ProRoot MTA, RMTA: Root MTA and CEM: experimental CEM cement).

The three negative control samples showed no evidence of dye penetration, whereas all three positive controls showed total dye penetration between gutta-percha and dentinal walls. All experimental groups demonstrated dye penetration, but this penetration was not beyond the root-end filling materials. The lowest dye penetration mean was observed in CEM cement root-end filled group (1.247± 0.915 mm), followed by RMTA (1.402 ± 1.017 mm), WMTA (1.705 ± 0.989 mm), and AMTA (1.720 ± 1,210 mm) root-end filled groups, respectively. The mean microleakage values are represented in Figure 1. The One-way ANOVA test was applied to the data. The results demonstrated no statistically significant differences in dye penetration among test groups.

## DISCUSSION

When a nonsurgical root canal treatment fails to treat periradicular lesions of endodontic origin, or retreatment is not indicated, endodontic surgery may be performed. The success of periradicular surgery is directly dependent to the achievement of a good apical seal, using a well-adapted root-end filling material. These materials are intended to prevent the leakage of potential irritants from the root canal system into the periradicular tissues (1-2).

Several methodologies have been employed to assess apical microleakage. They often include the use of dye/ink, bacterial/endotoxin leakage, radioisotope tracing and fluid filtration technique (3-10). There is no evidence in favor of superiority of any certain method. Dye penetration method is most popularly used for microleakage studies (26) because dyes are cheap, safe, available, and also relatively easy to be stored, used and to have their penetration evaluated quantitatively (27). Using animal models, dye leakage tests remain the best initial test for potential filling materials prior to in vivo experimentation (28).

The application of dye microleakage results, from laboratory studies, to clinical situation should always be considered carefully. However, if a root-end filling material does not allow penetration of small particles such as dyes, it is more likely to have the potential to prevent microleakage of bacteria and their by-products (29). In this study, linear measurement of methylene blue dye penetration was carried out as the criteria for judgment.

The purpose of this study was to compare the sealing capability of different MTAs as the most commonly used retrofilling material and CEM cement as a new root-end filling material. CEM cement group exhibited the lowest mean apical microleakage value; however, this difference was not statistically significant. We hypothesized that good sealing property of CEM cement can be explained by its handling characteristics and chemical properties. The mixed cement was not sticky and it did not adhere to applicator and allowed the operator to condense it easily. In addition, some calcium compounds such as calcium sulfate and calcium silicate help to a slight expansion of the material through continuous hydration after initial setting of the material and further crystalline maturation. Other studies are in progress to verify that this experimental cement possess other favorable properties i.e. biocompatibility, appropriate setting time, and suitable chemical properties.

As it was mentioned earlier, slight differences were found between commercial brands of MTAs (17,20,25). Such differences might be responsible for obtaining various amounts of dye microleakage. In spite of the differences, none of the roots in the experimental groups showed microleakage beyond the root-end filling material. This result is in agreement with various studies, which reported that MTA presented excellent sealing ability, and demonstrated its superiority in comparison with other commonly used root-end filling materials, using methylene blue as a dye (11,30).

## CONCLUSION

Based on the results obtained in this *in vitro* study, different commercial types of mineral trioxide aggregate (MTA) and calcium enriched mixture (CEM) cement as an experimental root-end filling material exhibited similar apical sealing ability.
